# Case Report: Bowel Occlusion Following the Use of Barbed Sutures in Abdominal Surgery. A Single-Center Experience and Literature Review

**DOI:** 10.3389/fsurg.2021.626505

**Published:** 2021-04-20

**Authors:** Guglielmo Stabile, Federico Romano, Davide De Santo, Felice Sorrentino, Luigi Nappi, Francesco Cracco, Giuseppe Ricci

**Affiliations:** ^1^Institute for Maternal and Child Health, Istituto di Ricovero e Cura a Carattere Scientifico “Burlo Garofolo”, Trieste, Italy; ^2^Department of Medical and Surgical Sciences, Institute of Obstetrics and Gynecology, University of Foggia, Foggia, Italy; ^3^University Clinical Department of Medical, Surgical and Health Sciences, University of Trieste, Trieste, Italy

**Keywords:** barbed suture, bowel occlusion, laparoscopy, hernia surgical repair, myomectomy

## Abstract

**Introduction:** A high level of surgical ability is required to perform endoscopic knot tying. Barbed sutures help in avoiding this procedure, thus reducing intraoperative time and lowering blood loss and hospitalization time when compared to traditional sutures. Some cases of bowel occlusion following the use of barbed sutures have been described in literature. All of them are characterized by the entanglement of an intestinal loop in wire barbs with bowel occlusion symptoms.

**Case Presentation:** We report two more cases which occurred in our Institute in 2020 and review those which have been reported in the literature by searching on Pubmed, Scopus, and Embase. We used the search terms: “Barbed,” ”Suture,” “Bowel,” and ”Obstruction.” We examined in the literature the surgical procedures, the type of complications, the time to onset of the complications, and the type of barbed suture.

**Discussion:** Twenty-two cases in total were reported in the literature from 2011 to 2020, and bowel complications were largely subsequent to interventions such as hernia surgical repair and myomectomy. In order to take advantage of barbed sutures while minimizing the risk of adverse events, such as intestinal occlusion, some precautions may be considered, such as the shortening of thread tails and use of antiadhesive barriers. Moreover, performing a few stitches backwards when ending the suture might be a useful suggestion. Further studies in this field may be useful in order to assess whether it might be better avoiding barbed suture application on serosal tissues to prevent bowel damage.

## Introduction

It is well-established that laparoscopic suturing with knot tying requires a substantial level of surgical skills, causing a significant increase in terms of operative time. Moreover, diverse animal studies showed that the knot tension obtained when performed by endoscopic means is lower than that obtained with traditional sutures (1). This fact may lead to an augmented risk of postoperative bleeding, hematomas, and cuff dehiscence (2, 3). After two cases of complications occurred in our hospital, we decided to investigate the pros and the cons of this relatively new type of knotless surgical thread.

Barbed sutures are nowadays widely used both in general surgery and gynecological procedures, and have been in use since the early sixties. Currently, different types of barbed suture are available on the market: Quill™ knotless tissue closure device (Angiotech™, Vancouver, BC, Canada), V-loc™ by Covidien™ (Covidien™, Mansfield, MA, USA), and Stratafix™ by Ethicon™ (Ethicon™, Cincinnati, OH, USA). All of them present barbs oriented in the opposite direction of the needle, which do not allow the suture to slide back (4).

The objective of this study is to provide an objective overview of the complications which may occur following the use of barbed sutures, considering all the benefits and disadvantages of this kind of wire. In the conclusion, some suggestions which may help avoiding adverse events are described.

## Case Description

### Case 1

In January 2020 a 32-year-old woman was admitted to the emergency room with nausea, vomiting, constipation, and abdominal pain. The woman underwent laparoscopic myomectomy 7 weeks before and had a history of multiple admissions to the emergency room for the same symptoms. Every time she had medical treatment, no pathologic clinical findings were diagnosed after clinical evaluation, ultrasonography, blood test, and X-rays. Meglumine diatrizoate (Gastrografin™) was administered to pursue bowel disobstruction, with no resolution of the symptomatology.

**Figure 1:**
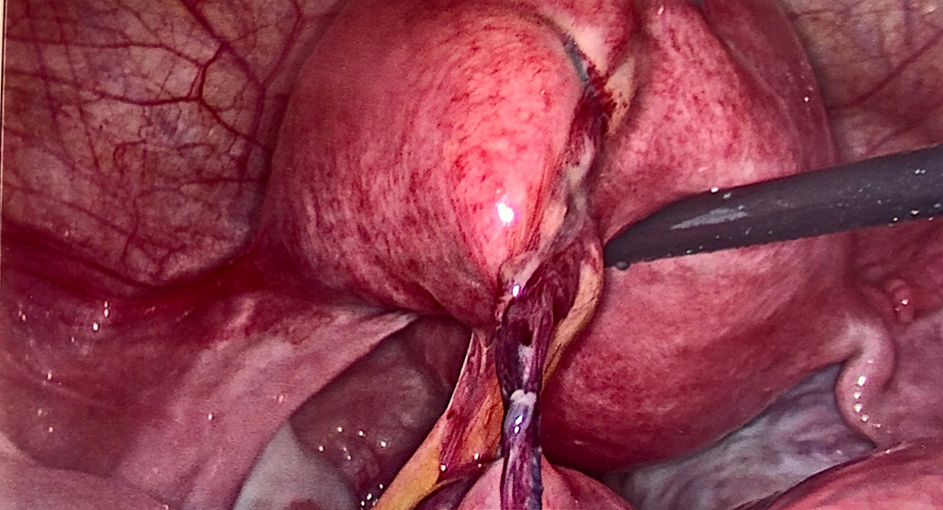
A tail of the absorbable barbed suture entangled in an ileal loop.

Considering the symptoms and the previous history of the woman, the decision was made to perform an urgent diagnostic laparoscopy. The scenario was the following: a tail of the absorbable barbed suture (V-Loc 90™ filament by Covidien™) which had been used to suture the uterine wound after the hysterotomy was entangled in an ileal loop, hence causing a subocclusion of the patient's small intestine. In addition to that, another V-Loc™ tail was entangled in the mesum close to the ileocecal valve (Figure 1). The filaments were promptly removed, and no resection of the small intestine was needed as the normal vascularization of the ansae was rapidly restored. The woman was discharged from the hospital 5 days after the surgery in good general condition. At the 6-month follow-up evaluation, no complications were reported (Table 1).

**Table 1:** Summary of case 1.

**Background**	**Presentation**	**Evaluation**	**1st line treatment**	**2nd line treatment**	**Surgical findings**	**Outcome**
32-year-old female, laparoscopic myomectomy 7 weeks before. Multiple admissions to the ER for nausea and constipation	Admission to ER with nausea, vomiting, constipation, and abdominal pain	Blood test, ultrasound, and X-rays did not corroborate suspected bowel occlusion	Gastrografin™ was administered with no resolution	Exploratory laparoscopy with removal of elongated barbed suture tail and bowel release	Tail of the absorbable barbed suture (V-Loc 90™) entangled in an ileal loop	Hospital discharge 5 days after surgery

### Case 2

At the end of the month of January 2020, a 76-year-old woman underwent surgery for vault prolapse. The surgeons' intention was to perform laparoscopic colposacropexy, but due to the intraoperative finding of a bone callus over the sacrum which had not been diagnosed before, colposacropexy could not be accomplished. Since the peritoneum covering the sacral bone had already been dissected, its closure was performed by the use of V-Loc 90 absorbable barbed suture. A laparoscopic lateral suspension was then achieved in order to treat the patient's prolapse. After 4 weeks the woman was admitted to the ER for bowel occlusion symptoms. Blood tests, electrocardiogram, X-rays, and CT were performed. A radiologic finding of intestinal volvulus emerged. Due to the poor clinical condition, a mini-laparotomy was then performed, and an entanglement of an intestinal ansa over the barbed suture tail was observed. Barbed suture wire detachment and trimming was carried out. The procedure was completed with volvulus untwisting and adhesiolysis. No bowel resection was needed. The woman was then discharged 3 days after the procedure. At the 6-month follow-up evaluation, no complications were reported (Table 2).

**Table 2:** Summary of case 2.

**Background**	**Presentation**	**Evaluation**	**Surgical findings**	**Treatment**	**Outcome**
76-year-old woman, laparoscopic correction of vault prolapse 4 weeks before	Admission to ER with nausea, vomiting, and constipation	Blood test, EKG, X-rays, and CT were performed. CT finding of intestinal volvulus	Entanglement of an intestinal ansa over a peritoneal barbed suture tail	Barbed suture wire detachment and trimming, bowel release	Hospital discharge 3 days after surgery

## Discussion

A review of the literature was performed by searching on Pubmed, Scopus, and Embase. We used the search terms: “Barbed,” ”Suture,” “Bowel,” and ”Obstruction.” We examined in the literature the surgical procedures, the type of complications, the time to onset of the complications, and the type of barbed suture. English only articles were included. The two patients involved in our manuscript signed their informed consent for the use of their data. The present study was designed according to the Helsinki declaration ethical principles. This retrospective observational descriptive study was approved by our Institutional Review Board (IRB-Burlo RC 08/2020).

Twenty cases of bowel occlusion have been described in the literature, from 2011 to 2020 (Table 3). The majority of them occurred following hernia surgical repair (30%, *n* = 6), alone or combined with other surgical techniques. After that, myomectomy (20%, *n* = 4) represents the second most frequent surgery causing this complication. Other less common causes are hysterectomy or sacrocolpopexy (15% each). Mean presentation time was 25 days (ranging from 1 to 210 days). Symptoms most commonly reported were abdominal pain, nausea, and vomiting. Less reported conditions were diarrhea and peritonitis. In 75% of cases (*n* = 15), resolution was achieved by laparoscopic ansae disentanglement; the remaining 15% (*n* = 5) underwent laparotomic surgery.

**Table 3:** Review of the literature.

**References**	**Journal and number of patients (*n*)**	**Procedure performed**	**Type of suture**	**Presentation, time**	**Treatment**
Donnellan et al. (5)	Journal of Minimally Invasive Gynecology, *n* = 1	Hysterectomy	Quill™, absorbable	Abdominal pain and vomiting, 30 days	Exploratory laparoscopy with barbed suture detachment and trimming
Thubert et al. (6)	International Urogynecologic Journal, *n* = 1	Sacrocolpopexy	V-loc™, absorbable	Abdominal pain and symptoms of bowel obstruction, 4 weeks	Midline laparotomy with adhesiolysis and obstruction release
Buchs et al. (7)	Minimally Invasive Therapy and Advanced Technologies, *n* = 1	Promontofixation, inguinal hernia repair, and pelvic floor repair	V-loc™, absorbable	Diffuse abdominal pain and vomiting, 8 days	Diagnostic laparoscopy with barbed suture trimming and bowel release
Kindinger et al. (8)	Gynecological Surgery, *n* = 1	Myomectomy	V-loc™, absorbable	Abdominal pain and distension, and loss of appetite, 4 weeks	Diagnostic laparoscopy with release of obstruction
Rombaut et al. (9)	Gynecological Surgery, *n* = 1	Myomectomy	Quill™, unspecified	Abdominal pain and diarrhea, paralytic ileus, 3 weeks	Diagnostic laparoscopy with barbed suture removal and disentanglement
Burchett et al. (10)	Journal of Laparoendoscopic and Advanced Surgical Techniques, *n* = 1	Myomectomy	V-loc™, absorbable	Severe abdominal pain and cramping, 4 weeks	Emergent exploratory laparotomy with volvulus reduction
Filser et al. (11)	International Journal of Surgical Case Reports, *n* = 1	Bilateral inguinal hernia repair	V-loc™, absorbable	Abdominal pain, 3 days	Exploratory laparoscopy with adhesiolysis and removal of suture wire
Köhler et al. (12)	Hernia, *n* = 1	Laparoscopic transabdominal preperitoneal hernia repair	V-loc™, unspecified	Small bowel obstruction, 13 days	Exploratory laparotomy with adhesiolysis and resection of redundant suture
Lee and Wong (13)	International Journal of Surgery Case Reports, *n* = 1	Myomectomy	V-loc™, absorbable	Acute peritonitis, 6 weeks	Emergent laparoscopy with adhesiolysis, release of barbed suture from rectum, excision of redundant suture over uterus, and peritoneal washing
Oor et al. (14)	Asian Journal of Endoscopic Surgery, *n* = 1	Laparoscopic roux-en-Y gastric bypass	V-loc™, absorbable	Abdominal pain and vomiting, 7 days	Diagnostic laparoscopy with removal of free barbed suture end
Segura-Sampedro et al. (15)	Revista espanola de enfermedades digestivas, *n* = 2	Rectopexy	V-loc™, unspecified	Diffuse abdominal pain and distension, 10 days	Exploratory laparotomy with strangulated bowel resection and double-barreled jejunoileosotmy
		Jejunostomy	V-loc™, absorbable	Abdominal pain, distension and vomiting, 2 days	Exploratory laparoscopy with release of adherent suture
Vahanian et al. (16)	Female Pelvic Medicine and Reconstructive Surgery, *n* = 2	Hysterectomy	V-loc™, unspecified	Abdominal pain and projectile vomiting, 22 days	Diagnostic laparoscopy with removal of elongated barbed suture tail and bowel release
		Hysterectomy	V-loc™, unspecified	Abdominal pain and vomiting, 4 weeks	Diagnostic laparoscopy with removal of elongated barbed suture and bowel release
Chen et al. (17)	Taiwan Journal of Obstetrics and Gynecology, *n* = 1	Hysterosacropexy	V-loc™, unspecified	Diffuse abdominal pain and vomiting after meals, 2 days	Diagnostic laparoscopy with release of redundant V-loc™ suture
Jang et al. (18)	Annals of Surgical Treatment and Research, *n* = 1	Gastrectomy	V-loc™, absorbable	Abdominal pain and distension, 4 days	Exploratory laparoscopy with complete closure of hernia and removal of surgical clip
Lee and Yoon (19)	Journal of Laparoendoscopic and Advanced Surgical Techniques, *n* = 1	Hepatico- jejunostomy	V-loc™, unspecified	Presentation unknown, 7 months	Hepaticojejunostomy revision
Tagliaferri et al. (20)	Journal of Surgery Case Report, *n* = 1	Laparoscopic transabdominal preperitoneal hernia repair	V-loc™, unspecified	Diffuse abdominal pain and distension, vomiting after eating, 1 day	Exploratory laparoscopy with redundant suture trimming and volvulus detorsion
Sartori et al. (21)	Il Giornale di Chirurgia, *n* = 1	Transabdominal hernia repair	V-loc™, absorbable	Abdominal pain and vomiting, 3 days	Diagnostic laparoscopy, wire cut and small bowel release
Zipple et al. (22)	The American Surgeon, *n* = 1	Laparoscopic inguinal hernia repair	V-loc™, absorbable	Abdominal pain, vomiting, and mild leukocytosis, 1 day	Exploratory lower midline laparotomy with removal of elongated barbed suture and bowel release

Due to the development of mini-invasive surgery in the last decade (23), barbed sutures' usage during surgical procedures is becoming more and more common, thus allowing young surgeons to avoid knot tying which represents one of the most difficult surgical steps. A range of studies have been conducted to assess whether the barbed suture is better than the traditional one in abdominal and pelvic surgery (24). In minimally invasive laparoscopic surgeries, the ability to quickly and properly tie surgical knots has presented a new challenge. In cases in which knot tying is difficult, the use of knotless barbed sutures can securely reapproximate tissues with less time, cost, and aggravation (25). Zhang et al. (26) in 2016 highlighted that barbed wires have the ability to reduce operative time both in laparoscopic myomectomy and mini-laparotomic myomectomy. In a 2020 meta-analysis (27), Wiggins et al. found that the use of barbed sutures for gastrointestinal anastomosis appears to be associated with shorter overall operative times, and no difference in rates of complications (including anastomotic leak, bleeding, or stricture) emerged when compared with standard suture materials. The suturing time decrease is attributable to multiple reasons, such as there being no need to tie knots nor to keep tension in the suture, due to the wire self-anchoring intrinsic feature (suture resists to migration) (28). Moreover, barbed sutures reduce total operative time and total amount of intraoperative blood loss when compared with traditional sutures (29). As proven by multiple studies, these observations are valid not only for gynecological procedures, but for other surgical areas too. Another characteristic of barbed sutures is the ability to maintain an equal distribution of tensile strength along the suture (28), which may be responsible for the avoidance of intramural hematomas formation. These, together with lowering the blood loss, may explain the reduction of post-operative patient's hemoglobin drop that has been observed (30). Surgery technical complexity is reduced too, as it is widely believed that laparoscopic sutures are one of the most difficult and time-consuming tasks during endoscopic surgery (29, 30). Finally, perioperative complications such as nosocomial infections or organic stressed events are diminished, as well as hospital stay; shorter hospitalization positively affects patients' physical and emotional quality of life, and may reduce hospital assistance costs too (31, 32).

Despite all the previously mentioned benefits, barbed suture-related complications must be carefully considered. The risk of bowel obstruction or intestinal volvulus is a significant downside to barbed suturing. A potential explanation of the occurrence of this kind of adverse event is the entanglement of barbed wires tails between intestinal ansae or mesa. Cutting barbed filament tails short enough may contribute to lessening this risk (33–35). Suture peritonization may not represent a valid solution since it does not avoid adhesion formation, as demonstrated by Api et al. in rats (36). The cases we reported in this study are the 21st and 22nd described in the literature by the time this article was written. These are the only two cases out of more than 400 surgeries accomplished in our Institute in which barbed sutures have been used (overall complication risk: 0.5%). Focusing just on gynecological procedures, our cases are part of fewer than 15 cases of bowel obstruction ever described following the usage of barbed sutures. This must be related to the fact that barbed sutures are increasingly executed in the gynecological field due to their ease of use and their efficacy in preventing hematoma formation and blood loss. As shown in Table 3, most of the cases of intestinal occlusion following the use of barbed sutures occurred after laparoscopic myomectomy (Table 3). This fact may be due to the close contact of intestinal loops with the barbed wires on the uterine wound. Other less common cases occurred after hysterectomy, and were related to entanglement of barbed sutures on the vaginal vault with the ansae. Furthermore, intestinal occlusion may happen following sacrocolpopexy, with adhesion formation between the ansae or mesum, and those wire barbs which protrude from the peritoneal suture. The cases we described belong to these categories.

**Figure 2:**
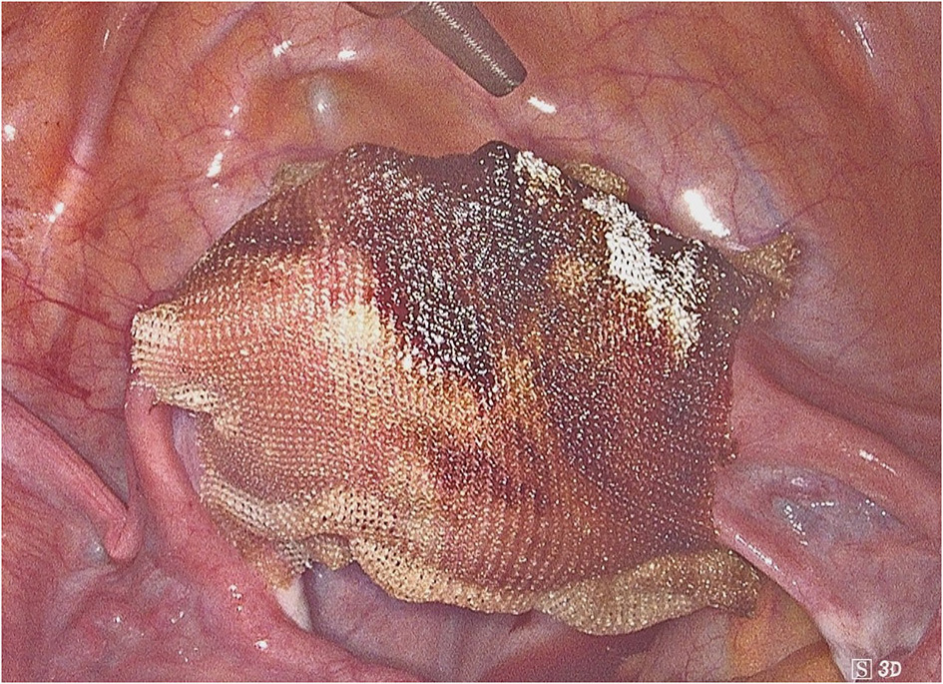
Adhesion barriers to avoid direct bowel contact with the barbed suture.

**Figure 3:**
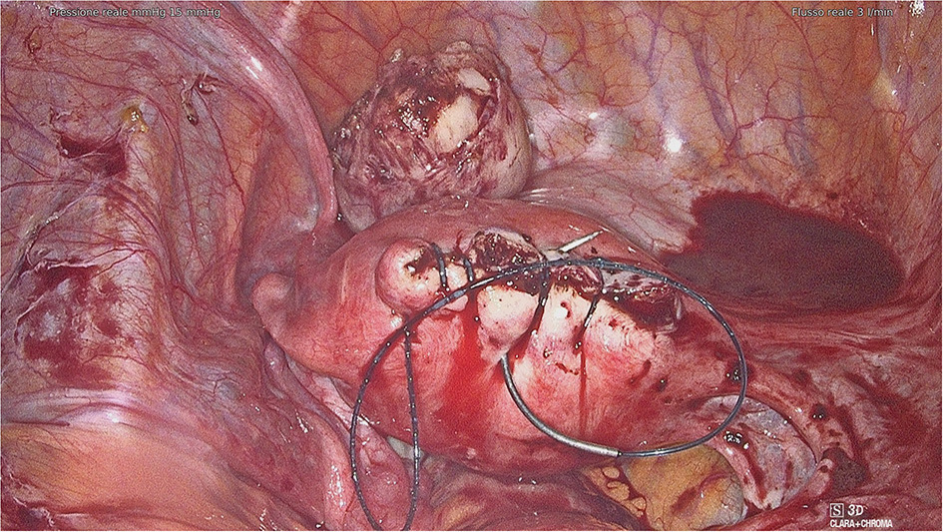
Tighten the suture by one or more backwards passages.

After a careful analysis of our cases and those reported in the literature, we propose to perform a second non-barbed suture above the barbed one in order to minimize complication risk. The closure of the uterine breach after myomectomy might be the procedure of choice when applying this precaution, as it often requires the execution of several suturing layers. This procedure may ensure a better tissue hemostasis and may reduce suture thread exposure too (Supplementary Video 1). Unfortunately, our suggestion partly decreases barbed suture advantages in terms of operating time. Moreover, on some occasions it might not be possible to perform a double layer suture, especially on serosa or peritoneum. Hence, if this expedient cannot be accomplished, adhesion barriers may be placed to avoid direct bowel contact with the barbed suture (Figure 2). Another trick that might help the surgeon to avoid excessive tail exposure is performing a few stitches backwards when ending the suture. Tissue shrinkage may lead to an overexposure of the barbed suture, and this must be kept in mind, especially when the suture is performed on myometrium after myomectomy. Tightening the suture by one or more backwards passages might avoid these problems too (Figure 3, Supplementary Video 2).

The weakness of our study is that is based largely on case reports, which do not provide solid solutions to barbed suture disadvantages. It is clear that more studies need to be done in order to understand how barbed sutures can be safely used, enjoying their benefits while minimizing the risk of complications.

Barbed sutures are an efficient device to minimize operative time, blood loss, and hospitalization time, which is of course related to the patient's recovery time. It is also an important aid for young surgeons with less experience in endoscopic surgery and laparoscopic knot tying (29). However, complications must be avoided. We summarized some precautions a good surgeon should consider when using barbed sutures: (1) performing one or two backwards stitches when ending the suture, in order to minimize the risk of suture slippage; (2) trying to use barbed sutures for intra-organ knots only (i.e., for deeper myometrial layers); (3) preferring absorbable non-barbed wires for serosal layers; (4) shortening thread free tails, especially when the suture is performed on myometrium after myomectomy (due to tissue shrinkage); and (5) applying long-lasting adhesion barrier devices when external barbed suture is inevitable. Development of long-lasting adhesion barriers may be a valid prospect for improving barbed suturing. More studies need to be done to understand how the use of this device can be optimized in surgical procedures.

## Data Availability Statement

The original contributions presented in the study are included in the article/Supplementary Materials, further inquiries can be directed to the corresponding author.

## Ethics Statement

The studies involving human participants were reviewed and approved by the Institutional Review Board of IRCCS BURLO GAROFOLO (RC 08/2020) approved this retrospective observational descriptive study. The patients/participants provided their written informed consent to participate in this study. Written informed consent was obtained from the relevant individual(s), and/or minor(s)' legal guardian/next of kin, for the publication of any potentially identifiable images or data included in this article. All patients signed an informed consent form before treatment. Permission for the publication was taken in accordance with the 1964 Helsinki Declaration and its later amendments or comparable ethical standards.

## Author Contributions

GS, FR, DD, and GR: conceptualization. GS, FC, DD, and FR: methodology. FC: software. FS and LN: validation. GS, FC, and FS: formal analysis. GS, FR, and FS: investigation. FC, GS, DD, and LN: data curation. GS and FC: writing original draft preparation. GR, FR, LN, and FS: writing, review, and editing. GR and LN: supervision. GR, LN, and GS: project administration. All authors have read and agreed to the published version of the manuscript.

## Conflict of Interest

The authors declare that the research was conducted in the absence of any commercial or financial relationships that could be construed as a potential conflict of interest.

## Abbreviations

ER: emergency room
EKG: electrocardiogram
CT: computed tomography.
